# A visual marker for early atrophy of the supraspinatus muscle on conventional MRI: introduction of the blackbird sign

**DOI:** 10.1007/s00330-024-10946-7

**Published:** 2024-07-11

**Authors:** Georg C. Feuerriegel, Roy P. Marcus, Sophia S. Goller, Adrian A. Marth, Karl Wieser, Samy Bouaicha, Reto Sutter

**Affiliations:** 1 Department of Radiology, Balgrist University Hospital, Faculty of Medicine, University of Zurich, Zurich, Switzerland; 2 Swiss Center for Musculoskeletal Imaging, Balgrist Campus AG, Zurich, Switzerland; 3 Department of Orthopedics, Balgrist University Hospital, University of Zurich, Zurich, Switzerland

**Keywords:** Rotator cuff, Muscular atrophy, Magnetic resonance imaging

## Abstract

**Objectives:**

The aim of this study was to introduce the blackbird sign as a fast, qualitative measure of early supraspinatus (SSP) muscle atrophy and to correlate the sign with quantitatively assessed muscle volume and intramuscular fat fraction (FF) in patients with full-thickness SSP tears.

**Materials and methods:**

The blackbird sign describes the asymmetric pattern of early SSP atrophy: on sagittal MR images, the supero–posterior contour of the muscle becomes concave, resembling the shape of a blackbird. MRIs of patients with full-thickness SSP tears were retrospectively reviewed for the presence of the blackbird and tangent signs. Patients were then divided into group 1: negative tangent sign and negative blackbird sign (*n* = 67), group 2: negative tangent sign and positive blackbird sign (*n* = 31), and group 3: positive tangent sign (*n* = 32). A 2-point Dixon sequence was acquired in all patients from which quantitative FF and muscle volumes were calculated.

**Results:**

In total 130 patients (mean age 67 ± 11 years) were included. Mean SSP volume was significantly smaller in group 3 (15.8 ± 8.1 cm^3^) compared to group 2 (23.9 ± 7.0 cm^3^, *p* = 0.01) and group 1 (29.7 ± 9.1 cm^3^, *p* < 0.01). Significantly lower muscle volumes were also found in group 2 compared to group 1 (*p* = 0.02), confirming that the blackbird sign is able to identify early SSP atrophy. Mean FF in the SSP was significantly higher in group 3 (18.5 ± 4.4%) compared to group 2 (10.9 ± 4.7%, *p* < 0.01) and group 1 (6.1 ± 2.6%, *p* < 0.01).

**Conclusion:**

Visual assessment of early muscle atrophy of the SSP is feasible and reproducible using the blackbird sign, allowing the diagnosis of early SSP atrophy.

**Clinical relevance statement:**

In routine clinical practice, the blackbird sign may be a useful tool for assessing early muscle degeneration before the risk of postoperative rotator cuff re-tears increases with progressive muscle atrophy and fatty infiltration.

**Key Points:**

- *Quantitative measurements of rotator cuff injuries require time, limiting clinical practicality*.
- *The proposed blackbird sign is able to identify early SSP atrophy*.
- *Reader agreement for the blackbird sign was substantial, demonstrating reproducibility and ease of implementation in the clinical routine*.

## Introduction

Rotator cuff (RC) tears are one of the most common causes of shoulder pain and functional impairment among individuals of all age groups [1, 2]. Accurate diagnosis and appropriate management of SSP tears are important, as it is the most commonly affected muscle in RC tears [3]. Following tendon rupture, degenerative changes occur in the RC muscle, including fatty infiltration and atrophy [3–6]. The degree of fatty muscle infiltration and atrophy has been shown to correlate with repairability and outcome of tendon-to-bone repair [7]. Various visual/qualitative and quantitative radiological techniques have been developed to aid in the diagnosis and assessment of SSP tears [8–13]. Among the most promising quantitative techniques for assessing RC fatty muscle infiltration are chemical shift-based methods and Dixon-based techniques, including extended 2-point Dixon methods [14–18]. However, quantitative assessments require the acquisition of special MR sequences, and their acquisition, postprocessing, and segmentation is time-consuming which limits their use in routine clinical practice.

Therefore, a rapid and reliable visual assessment of the SSP muscle is required, creating the need for qualitative and semi-quantitative scoring systems. One of the most commonly used classifications for assessing fatty infiltration has been described by Goutallier et al [9]. However, it has been noted that this classification can be influenced by medial retraction of the myotendinous unit in full-thickness tears and has limited inter-rater reliability, thereby reducing its usefulness in clinical routine [19, 20]. In addition, only the fatty infiltration of the muscle is assessed, while the effects of muscle atrophy are not evaluated separately.

Current methods to assess SSP muscle atrophy include the occupation ratio and tangent sign [12, 13, 21]. The tangent sign uses a reference line drawn between the superior borders of the spina scapulae and the coracoid process on the most lateral sagittal oblique MR image. When the SSP muscle undergoes atrophy, its belly falls below this line, which is considered a positive tangent sign and is associated with both increased fatty muscle infiltration and a worse outcome after RC repair [22, 23]. Thomazeau et al established the use of an occupation ratio which is calculated from the surface of the SSP muscle divided by the surface of the entire SSP fossa [21]. However, both methods have limited capabilities to assess early atrophy of the SSP muscle and, moreover, consider muscle atrophy to be a symmetric, respectively homogeneous process throughout the muscle belly. In clinical routine, we have often encountered cases of SSP tendon pathology where the tangent sign is negative, but where the supero–posterior contour of the SSP muscle belly is concave, rather than convex, resembling the shape of a blackbird on sagittal MR images.

To assess this perception, this study evaluated the blackbird sign as a fast, qualitative sign of early SSP muscle atrophy and correlated this sign with quantitatively assessed muscle volume and intramuscular fat fraction (FF) in patients with full-thickness SSP tendon tears.

## Materials and methods

### Patient selection

**Fig. 1:**
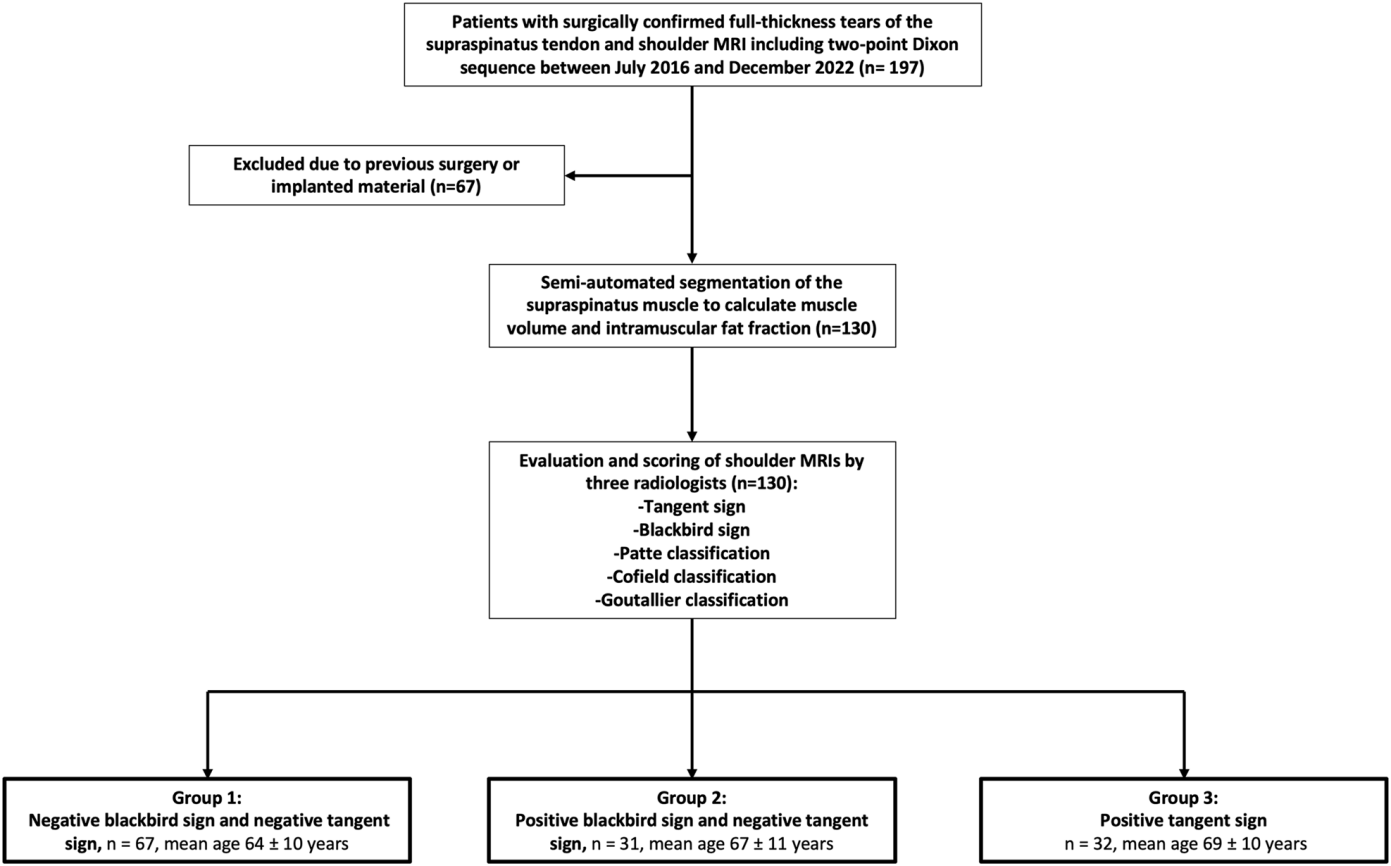
Patient selection flow chart. Patients with surgically confirmed supraspinatus (SSP) tears were divided into three groups according to the results of the MR-based assessments. Group 1: patients with a negative tangent sign and negative blackbird sign (*n* = 67). Group 2: patients with positive blackbird sign but a negative tangent sign, i.e., early SSP muscle atrophy (*n* = 31). Group 3: patients with a positive tangent sign, i.e., advanced SSP muscle atrophy (*n* = 32)

Patient records were retrospectively reviewed for diagnosis of a full-thickness tear of the SSP tendon, and patients were eligible if they underwent shoulder MRI including a 2-point Dixon sequence between July 2016 and December 2022 (*n* = 197, Fig. 1). Inclusion was also possible in the case of additional tears of the infraspinatus tendon and the subscapularis tendon. Patients with inflammatory rheumatoid arthritis, previous surgery for RC reconstruction, fracture, or osteoarthritis were excluded. All patients underwent surgical RC repair, confirming full-thickness SSP tears. Morphological MR images were assessed and graded by three radiologists (R.P.M. with more than 10 years of experience, S.S.G. with more than 5 years of experience and A.A.M. with more than 4 years of experience in radiology). From the 2-point Dixon sequence, FF maps were reconstructed on the console, which was then semi-automatically segmented to obtain intramuscular FF and muscle volume of the SSP. Patients were split into three groups for evaluation. Patients with negative tangent signs and negative blackbird signs were included in group 1 (*n* = 67). Patients with positive blackbird sign but negative tangent sign were included in group 2 (*n* = 31), and patients with positive tangent sign only were included in group 3 (*n* = 32). The study was approved by our institutional review board (Cantonal Ethics Committee Zurich). Written informed consent was obtained from all study participants.

### MR imaging

MR imaging of the shoulder was performed on a 3-T scanner (Magnetom Skyrafit or Magnetom Vida; Siemens Healthcare) with a dedicated 16-channel shoulder coil. All patients underwent a routine shoulder imaging protocol including a sagittal oblique 2-point Dixon sequence from which water-only, fat-only, in-phase and out-of-phase images were acquired (detailed scan parameters are listed in Table 1). Signal intensity values on in-phase and fat-only images were defined as S(In) and S(Fat). FF maps were calculated using the following equation as previously described [16, 18, 24]:

$$	\,\,\,{{{{{\rm{S}}}}}}({{{{{\rm{Water}}}}}})+{{{{{\rm{S}}}}}}({{{{{\rm{Fat}}}}}})={{{{{\rm{S}}}}}}({{{{{\rm{In}}}}}})\\ {{{{{\rm{Fat}}}}}}\; {{{{{\rm{fraction}}}}}}=\, 	{{{{{\rm{S}}}}}}({{{{{\rm{Fat}}}}}})/({{{{{\rm{S}}}}}}({{{{{\rm{Water}}}}}})+{{{{{\rm{S}}}}}}({{{{{\rm{Fat}}}}}}))={{{{{\rm{S}}}}}}({{{{{\rm{Fat}}}}}})/{{{{{\rm{S}}}}}}({{{{{\rm{In}}}}}})$$

**Table 1:** Sequence parameters for the 3-T MR imaging protocol

Sequence	Sagittal oblique 2-point Dixon	Coronal PD blade FD	Coronal T1 TSE FS	Sagittal oblique T2 FS blade	Sagittal oblique T1 TSE	Axial T2 TRUFI FS
Echo time, (ms)	1.31, 2.57	34	12	71	11	4.84
Repetition time, (ms)	6.69	2200	500	3800	450	11.2
Bandwidth, (Hz/px)	1010	220	200	120	200	200
Acquisition matrix	120 × 160	384 × 384	448 × 358	256 × 256	384 × 397	512 × 256
Slice thickness, (mm)	3	4	3	4	4	1, 7
Slice number	52	20	20	24	29	52
FOV, (mm)	160 × 160	159 × 159	160 × 160	160 × 160	159 × 159	180 × 180
Acquisition time, (min)	1.02	3.40	2.11	3.10	2.04	3.41

### Image analysis

SSP tendon tear size was graded according to Cofield et al and divided into: small (less than 1 cm tear size), medium (1–3 cm tear size), large (3–5 cm tear size), and massive (over 5 cm tear size) [10]. Tendon retraction was graded according to Patte et al as stage 1: proximal stump close to the bony insertion, stage 2: proximal stump at the level of the humeral head, and stage 3: proximal stump at the level of the glenoid [11]. Goutallier grading was used for visual assessment of fatty infiltration of the SSP muscle using the following grades: 0 = normal muscle, grade 1 = some fatty streaks, grade 2 = less than 50% fatty muscle atrophy, grade 3 = 50% fatty muscle atrophy, and grade 4 = more than 50% fatty muscle atrophy [9].

The tangent sign [12] and the newly proposed blackbird sign were used for visual assessment of SSP muscle atrophy.

**Fig. 2:**
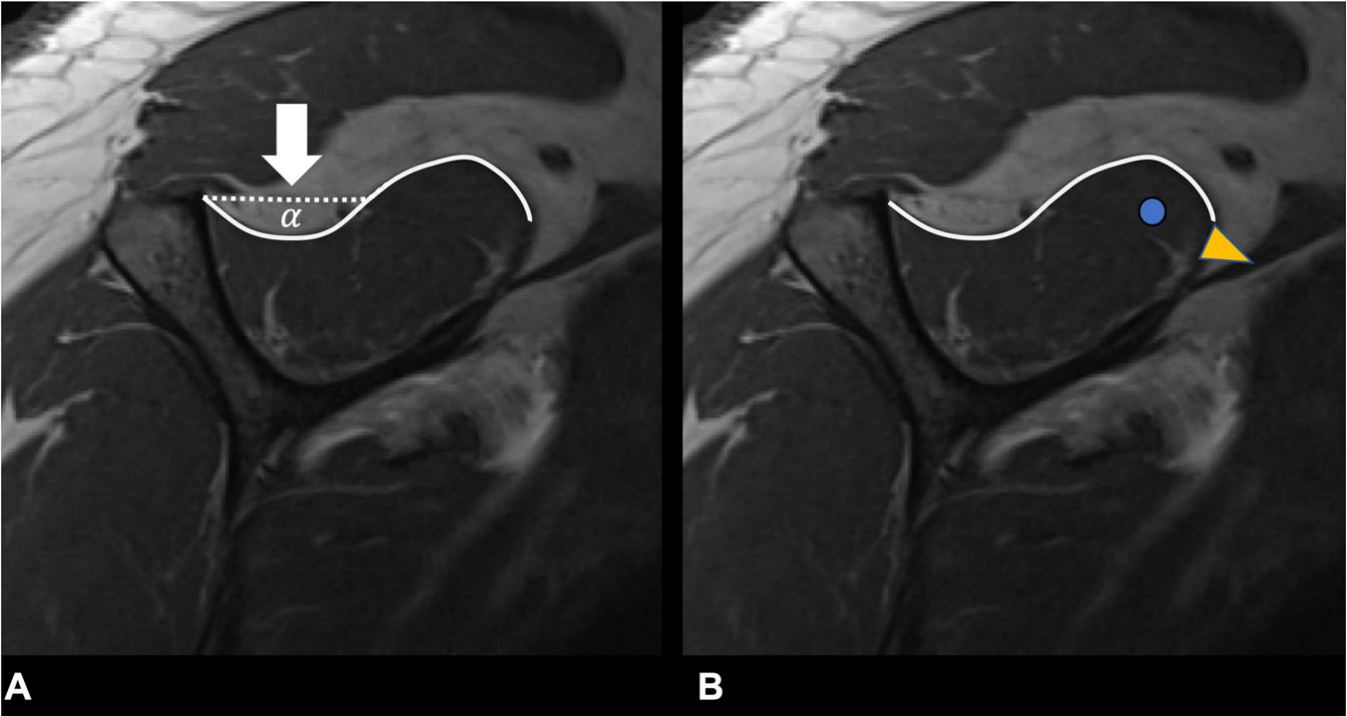
Sagittal oblique T1-weighted images of a 59-year-old patient with early SSP muscle atrophy resulting in a positive blackbird sign (**A**, **B**). The blackbird sign is assessed at the centre of the SSP muscle. The atrophy of the supero–posterior SSP muscle subregion creates an inwardly curved muscle contour, giving the impression of an anterior-facing blackbird. In contrast, the medial and inferior SSP muscle subregions appear unchanged, while the atrophied region is filled with fatty tissue (asterisk)

The blackbird sign was assessed on sagittal oblique T1-weighted images at the centre of the SSP muscle. The sign evaluates early volume atrophy of the supero–posterior parts of the SSP muscle, while no significant fatty infiltration has occurred. As a result, the SSP muscle loses its convex shape and the atrophied muscle parts create an inwardly curved contour, i.e., a focal concave shape that resembles a blackbird (Fig. 2). The medial and inferior parts of the SSP muscle belly appear unchanged, while the atrophied region appears to be occupied by fatty tissue (Fig. 2).

**Fig. 3:**
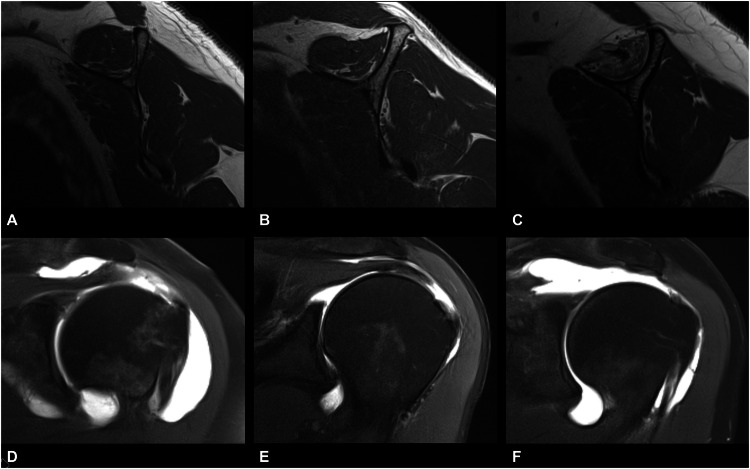
Sagittal oblique T1-weighted images of one patient from each group at the level of the middle of the SSP muscle belly (**A**–**C**) with their corresponding coronal fat-saturated proton density-weighted images showing the full-thickness SSP tears in the lower row (**D**–**F**). The normal convex shape of the SSP muscle is readily visible in the patient in group 1 (**A**) compared to the patient in group 2 with a positive blackbird sign (**B**) and the patient in group 3 with advanced muscle atrophy (**C**). Note also the advanced fatty SSP muscle infiltration and greater tendon retraction in the patient in group 3 (**C**, **F**)

**Fig. 4:**
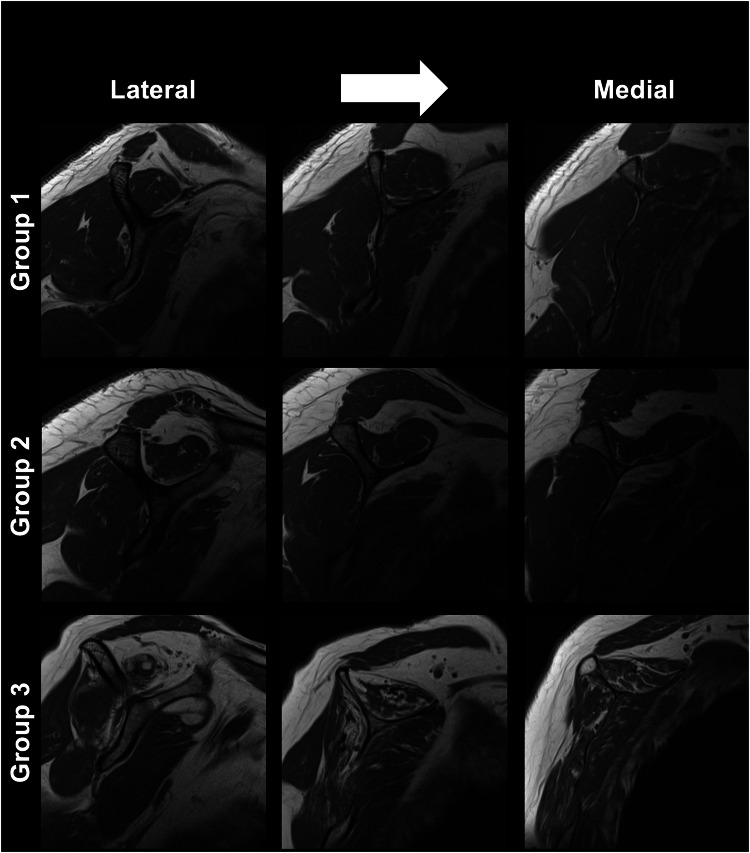
Multiple sagittal oblique T1-weighted images from one patient in each group, arranged from lateral (left) to medial (right). Group 1: normal SSP muscle. Group 2: early SSP muscle atrophy, i.e., positive blackbird sign (outline arrow). Group 3: advanced muscle atrophy, i.e., positive tangent sign. Note, that the change in the supero–posterior contour of the superior SSP muscle is due to early atrophy in the patient in group 2 with a positive blackbird sign. In addition, the patient with a positive tangent sign in group 3 also shows a substantial increase in fat infiltration compared to groups 1 and 2

To assess the tangent sign, a horizontal line was drawn from the upper edge of the scapular spine to the upper edge of the coracoid process in the most lateral position, with the “Y” of the scapula being visible. In a healthy muscle, the border of the SSP muscle should cross this horizontal line. If the muscle was below this line, it was considered a positive tangent sign representing advanced muscle atrophy (Figs. 3 and 4) [12]. The MR images were read individually and independently in random order and blinded to clinical information and any other imaging data. In the second step, the ratings were reviewed for consensus to ensure correct intergroup assessment. In case of disagreement, a fourth reader (blinded for review) was asked for a decision. Analysis was performed on a PACS workstation certified for clinical use (MERLIN 7.1.22, Phönix-PACS GmbH).

### Quantitative analysis

**Fig. 5:**
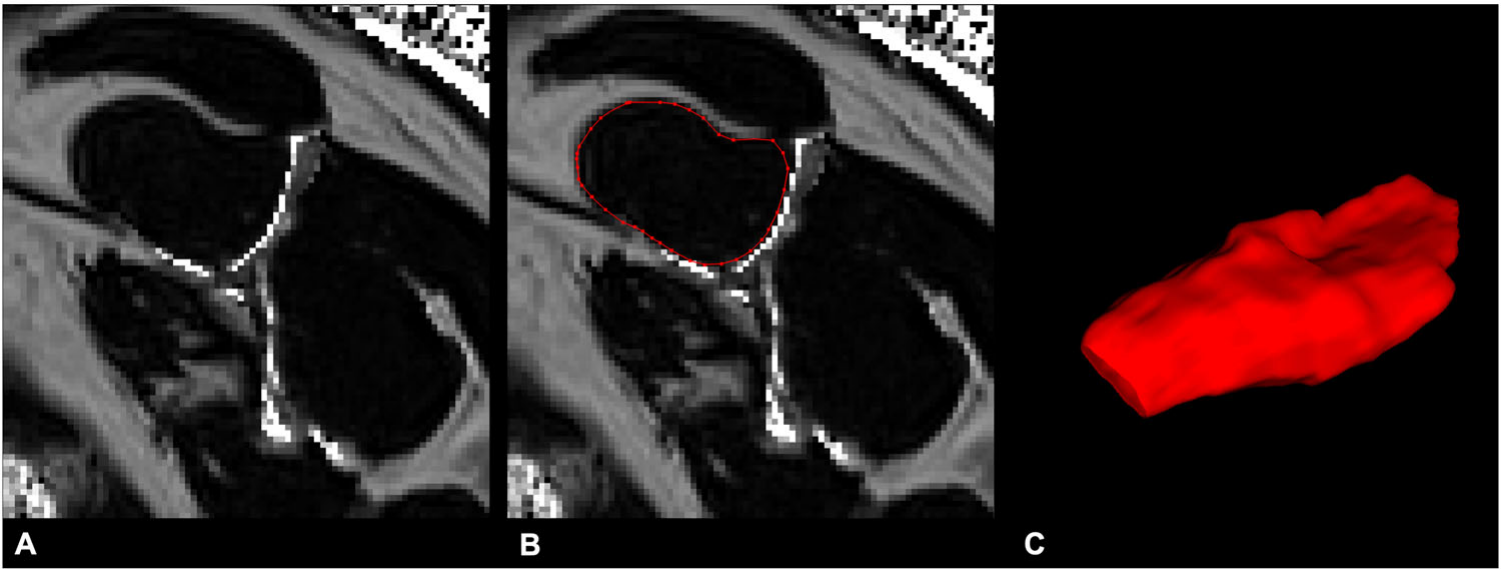
Sagittal oblique FF map of a 55-year-old patient calculated from the 2-point Dixon sequence (**A**). Semi-automated segmentation was performed by drawing regions of interest (ROIs) on every five slices around the SSP muscle (**B**). **C** Shows the 3D model of the segmented SSP muscle (red)

Quantitative analysis of the SSP muscle volume and FF was performed by G.C.F. using ITK snap [25]. Regions of interest (ROIs) were manually drawn on every 5th slide of the SSP muscle in the sagittal oblique plane of the FF images calculated from the 2-point Dixon sequences. Missing ROIs were semi-automatically interpolated by the segmentation software and then manually checked for correct placement. The total time for semi-automated segmentation, verification and analysis of quantitative values took approximately 5 min per patient. Mean muscle volume and FF of the SSP muscle were reported for each patient (Fig. 5).

### Statistics

Non-normal distribution of data was assessed using the Shapiro–Wilk test. In addition to descriptive statistics, the Kruskal–Wallis test with Bonferroni correction was used to assess differences in FF and muscle volume between groups. One-way ANOVA with Bonferroni correction was used to compare Patte, Cofield, and Goutallier scores between groups. Receiver operating characteristic (ROC) curves and the Youden index were used to identify cut-off values for FF and muscle volume that best predicted the blackbird and tangent sign. Inter- and intra-reader agreement was assessed using Fleiss’ Kappa [26]. Statistics were performed in SPSS (v. 28.0 IBM Corp.) under the supervision of an experienced biostatistician.

## Results

A total of 130 patients (mean age 67 ± 11 years, 79 women, Table 2) were included in the study. Sixty-seven patients with negative tangent sign and negative blackbird sign were included in group 1, 31 patients with positive blackbird sign and negative tangent sign were included in group 2. A positive tangent sign and a negative blackbird sign were observed in 32 patients included in group 3.

**Table 2:** Comparison of patient characteristics between the groups

	Group 1^d^	Group 2^e^	Group 3^f^	*p*-value^b^
Age, (years)^a^	64 ± 10	67 ± 11	69 ± 10	> 0.05
Female	43	17	19	
Full thickness SSP tear, (*n*)	67	31	32	
Cofield classification^a,c^	1.8 ± 0.6	2.0 ± 0.7	2.8 ± 0.8	< 0.01
Patte classification^a,c^	1.8 ± 0.5	1.9 ± 0.6	2.3 ± 0.5	< 0.01
Goutallier classification^a,c^	0.8 ± 0.5	1.4 ± 0.6	1.9 ± 0.8	< 0.01
Mean SSP muscle volume, (cm^3^)^a,c^	29.7 ± 9.1	23.9 ± 7.0	15.8 ± 8.1	< 0.01
Mean SSP muscle FF, (%)^a,c^	6.1 ± 2.6	10.9 ± 4.7	18.5 ± 4.4	< 0.01

*n* number of patients, *SSP* supraspinatus

^a^ Data is given as Mean ± standard deviation

^b^ Comparison between the groups using a one-way analysis of variance

^c^ Significant difference detected in the Bonferroni adjusted post hoc analysis

^d^ Group 1: patients with negative blackbird and tangent sign

^e^ Group 2: patients with positive blackbird and negative tangent sign

^f^ Group 3: patients with negative blackbird and positive tangent sign

Tendon retraction was rated significantly higher in group 3 (Patte: mean 2.3 ± 0.5) compared to group 2 (Patte: mean 1.9 ± 0.6, *p* = 0.01) and group 1 (Patte: mean 1.8 ± 0.5, *p* < 0.01). Similarly, the defect size of the SSP tendon was rated significantly larger in group 3 (Cofield: mean 2.8 ± 0.8) compared to group 2 (Cofield: mean 2.0 ± 0.7, *p* = 0.02) and group 1 (Cofield: mean 1.8 ± 0.6, *p* < 0.01). Tendon retraction and defect size were also rated higher in group 2 than in group 1, but this did not reach statistical significance (Patte: *p* = 0.98, Cofield: *p* = 0.63). Visual assessment of fatty muscle infiltration showed a significantly higher score in group 3 (Goutallier: mean 1.9 ± 0.8) compared to group 2 (Goutallier: mean 1.4 ± 0.6, *p* = 0.04) and group 1 (Goutallier: mean 0.8 ± 0.5, *p* < 0.01), but also in group 2 compared to group 1 (Goutallier: *p* < 0.01).

**Fig. 6:**
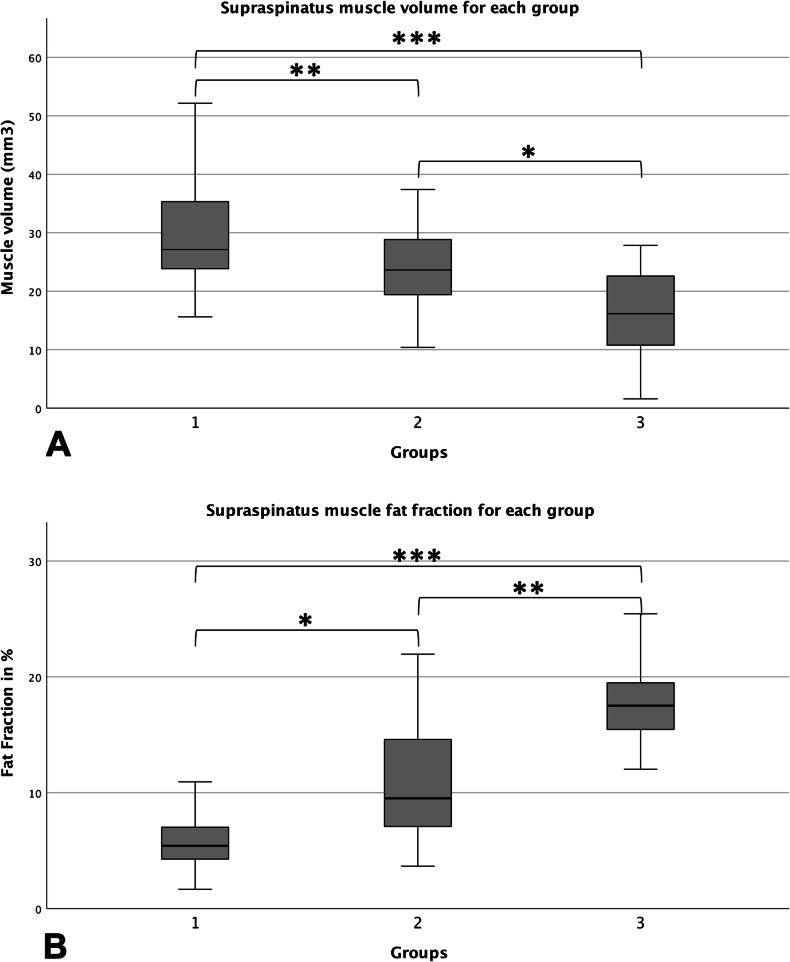
Box plots of SSP muscle volume (**A**) and intramuscular FF (**B**) calculated from the quantitative 2-point Dixon sequence for each group. Bonferroni adjusted intergroup assessment revealed significant differences between each group for the muscle volume, as well as the intramuscular FF (asterisks)

The visual assessment of fatty infiltration was confirmed by the quantitative assessment of FF of the whole segmented SSP muscle. The mean muscle FF of group 3 (mean FF: 18.5% ± 4.4) was significantly higher compared to group 2 (mean FF: 10.9% ± 4.7, *p* < 0.01) and group 1 (mean FF: 6.1% ± 2.6, *p* < 0.01). SSP muscle volume was significantly smaller in group 3 (mean volume: 15.8 cm^3^ ± 8.1) compared to group 2 (mean volume: 23.9 cm^3^ ± 7.0, *p* = 0.01) and group 1 (mean volume: 29.7 cm^3^ ± 9.1, *p* < 0.01). Significantly higher SSP muscle FF and lower muscle volume were also found in group 2 compared to group 1 (FF: *p* < 0.01, volume: *p* = 0.02). The differences in the quantitative assessment of muscle FF and volume between the groups are again illustrated in Fig. 6.

The ROC analysis revealed a good performance in discriminating the blackbird sign and the tangent sign using the muscle volume (area under the curve (AUC) 0.72 and 0.78), as well as the intramuscular FF (AUC 0.81 and 0.95).

Using sensitivity and specificity, the Youden index was calculated to define ideal cut-off values that best predict the blackbird and the tangent sign. The best performance was calculated for a muscle volume of 23.9 cm^3^ (sensitivity 0.781 and specificity 0.813) for the blackbird sign and 16.8 cm^3^ (sensitivity 0.844 and specificity 0.783) for the tangent sign. For the intramuscular FF, ideal cut-off values were calculated at an FF of 7.1% (sensitivity 0.870 and specificity 0.821) for the blackbird sign and 13.1% (sensitivity 0.938 and specificity 0.876) for the tangent sign.

The analysis of the inter-reader agreement between the three readers showed an overall substantial agreement for the detection of the blackbird sign (Fleiss’ Kappa: 0.76 [0.68–0.85]), as well as for the tangent sign (κ 0.72 [0.63–0.80], Table 3). A substantial agreement was also found for the assessment of the tendon defect, retraction and fatty muscle infiltration (Fleiss’ Kappa range: 0.71–0.82). Intra-reader analysis was performed by Reader 1 and 2 with at least 4 weeks in between readings: again, a substantial agreement was found for the blackbird sign for both readers (Reader 1: κ 0.71 [0.61–0.84], Reader 1: κ 0.75 [0.63–0.82], Table 3).

**Table 3:** Inter- and Intra-reader agreement for qualitative assessment of the SSP tendon and muscle

	Interreader^a^	Intrareader^b^	
Parameters		Reader 1	Reader 2
Goutallier classification	0.71 [0.62–0.79]	0.73 [0.65–0.82]	0.70 [0.61–0.79]
Patte classification	0.78 [0.65–0.82]	0.83 [0.74–0.91]	0.80 [0.72–0.87]
Cofield classification	0.81 [0.75–0.87]	0.78 [0.71–0.83]	0.81 [0.67–0.91]
Blackbird sign	0.76 [0.68–0.85]	0.71 [0.61–0.84]	0.75 [0.63–0.82]
Tangent sign	0.72 [0.63–0.80]	0.79 [0.71–0.89]	0.72 [0.62–0.79]

^a^ Fleiss’ kappa (κ) for Reader 1–3

^b^ Weighted Cohen’s kappa (κ)

## Discussion

In this study, we demonstrated the suitability of the blackbird sign as a fast, qualitative marker of early atrophy of the SSP muscle after full-thickness tendon tear. Compared to patients with a positive tangent sign, patients with a positive blackbird sign had a higher muscle volume and a lower intramuscular FF, indicating an earlier stage of muscle degeneration. In contrast, patients with both a negative blackbird and tangent sign had even higher muscle volumes and lower FFs than patients with a positive blackbird sign.

The tangent sign is the most established visual marker of SSP muscle atrophy. Assessing the upper edge of the muscle belly either below or above a horizontal line drawn from the upper edge of the scapula to the coracoid process is relatively simple and quick, and a positive sign has been found to correlate with poorer patient outcomes [22]. However, the tangent was also positively correlated with advanced fatty muscle infiltration (Goutallier > 2) and increased tear size, suggesting an advanced stage of muscle degeneration in the case of a positive tangent sign [27, 28]. The tangent sign can therefore not be used to assess early muscle atrophy. In addition, RC repair in patients with fatty muscle infiltration greater than Goutallier grade 2 is considered to have a higher risk of re-tears, and it is debatable to what extent these patients should be surgically treated at all, further limiting the clinical use of the tangent sign.

The blackbird sign proposed in this study assesses early signs of atrophy of the SSP muscle before advanced fatty muscle infiltration has occurred and may be helpful for surgical decision-making at an earlier stage: In a previous study, it was shown that the risk of re-tear after RC repair of the SSP muscle increases with a FF greater than 6% [29]. In our study, the blackbird sign was best predicted at a FF of 7.1%, which is much closer to the 6% threshold than the tangent sign (13.1%). Therefore, the blackbird sign might indicate the stage of muscle atrophy and fatty infiltration to the surgeon above which the risk of postoperative RC re-tears increases significantly without having to segment the whole muscle on quantitative sequences.

The second most common way to qualitatively assess SSP muscle atrophy is to calculate the occupation ratio between the surface area of the SSP muscle and the entire SSP fossa [21]. In contrast to the tangent sign, this method also allows the assessment of a lower degree of muscle atrophy, but requires manual alignment and measurement of the different surfaces, which is time-consuming and leaves room for error. In addition, the occupation ratio tends to overestimate SSP muscle atrophy in cases of full-thickness tears, due to the retraction of the myotendinous unit [30]. More importantly, the ratio method assumes that the SSP muscle belly atrophies in a concentric fashion. In contrast, a study by Meyer et al showed that the SSP muscle rather undergoes an asymmetric pattern of degeneration after tendon rupture [31]. They demonstrated that the superior parts of the muscle are more prone to atrophy, while the inferior parts of the muscle are more prone to fatty infiltration. Similar results were found in a study by Trevino et al in a cadaveric study, in which the authors divided the SSP muscle into subregions and quantitatively assessed the muscle volume [32]. They concluded that the superficial subregions of the SSP are mainly affected by muscle atrophy, whereas the deep subregions are mainly affected by fatty infiltration. While the two studies by Meyer and Trevino evaluated SSP atrophy, they did not subdivide their study collectively with a separate group with early atrophy. The results from our study confirm that there is a distinct population of patients with full-thickness SSP tears where early atrophy is present, and this presents as a focal concave shape of the supero–posterior aspect of the SSP muscle, resembling the shape of a blackbird. It is important to note that the blackbird sign can only be assessed if the tangent sign is negative, i.e., when there is not already advanced muscle atrophy.

Another observation made in this study was the significantly greater tendon retraction in patients with a positive tangent sign (*p* = 0.01). Previous studies have suggested that tendon retraction produces false positive tangent signs and that assessment should be performed more medially [33, 34]. However, in our study, we were not able to confirm these observations. Quantitative analysis showed that patients with positive tangent signs had significantly smaller muscle volumes and higher FFs suggesting that tendon retraction does not produce false negative tangent signs.

There are several limitations of this study that need to be addressed. First, the study design was retrospective, with patients being examined at a single centre, but in all patients, the full-thickness tears were surgically confirmed. The assessment of the blackbird sign was based solely on MR-morphological results, and no other imaging modality was available for confirmation. However, the correlation with 3D analysis of the muscle volume confirms the validity of our findings.

In conclusion, visual assessment of early muscle atrophy of the SSP in patients with full-thickness tendon tears is feasible and reproducible using the newly introduced blackbird sign. In routine clinical practice, the blackbird sign may therefore be a useful tool for assessing early muscle degeneration before the risk of postoperative RC re-tears increases with progressive muscle atrophy and fatty infiltration.

## Abbreviations

AUC: Area under the curve
FF: Fat fraction
RC: Rotator cuff
ROC: Receiver operating characteristic curve
ROI: Region of interest
SSP: Supraspinatus muscle
